# Concurrent anti-glomerular basement membrane disease and membranous nephropathy: a case report

**DOI:** 10.3389/fmed.2025.1654203

**Published:** 2025-11-04

**Authors:** Lin-Lin Li, Pei-Yuan Niu, Na Li, Xiao-Ling Zhang, Hui-Xia Cao

**Affiliations:** Renal Division, Henan Provincial People's Hospital, Zhengzhou University People's Hospital, Zhengzhou, China

**Keywords:** anti-glomerular basement membrane (GBM) disease, membranous nephropathy (MN), diagnosis, phospholipase A2 receptor (PLA2R), treatment, prognosis

## Abstract

Anti-glomerular basement membrane (GBM) disease is a rapidly progressive glomerulonephritis which, in rare instances, occurs concurrently with membranous nephropathy (MN). We report a case of this patient presented with proteinuria and hematuria, the predominant pathology was crescentic and necrotizing glomerulonephritis with linear staining for immunoglobulin G (IgG) along the glomerular basement membrane (GBM) associated with epi-membranous electron dense immune deposits. The patient tested had significant titers of anti-GBM antibodies (196.27 RU/ml; reference range:0–20 RU/ml), and renal biopsy demonstrated diffuse strongly positive granular staining for phospholipase a2 receptor (PLA2R) along the GBM. The patient received therapy including prednisone, plasmapheresis and CD20 antibodies, which resulted in the clearance of anti-GBM antibodies and eventual recovery of renal function, as evidenced by the return of serum creatinine to normal levels. Earlier diagnosis and timely effective treatments could improve patients' prognosis.

## Introduction

Anti-glomerular basement membrane (GBM) disease is a rapidly progressive glomerulonephritis which progresses to end stage kidney disease if not treated early and intensively. It is typically characterized by necrotizing and crescentic glomerulonephritis with linear deposits of immunoglobulin G (IgG) along the GBM, and some patients present with concurrent alveolar hemorrhage. Characterized by antibodies against the NC1 domain of IV collagen, anti-GBM disease is usually treated with a regimen of plasmapheresis, cyclophosphamide, and corticosteroids (1). Membranous nephropathy (MN), typically presents with proteinuria and nephrotic syndrome, is characterized by sub-epithelial immune deposits of IgG and complement along the glomerular capillary wall (2).

In clinical practice, anti-GBM disease occasionally co-exists with a second disease, among which the “mixed” phenotype with anti-neutrophil cytoplasmic antibody (ANCA) associated vasculitis (AAV) is relatively common, which have a truly hybrid disease phenotype, requiring aggressive early treatment for anti-GBM disease, and careful long-term follow-up and consideration for maintenance immunosuppression for AAV (3). In contrast, the coexistence of anti-GBM disease and MN is extremely rare. Primary MN typically does not present with crescents; the presence suggests a secondary disease, for example lupus nephritis with combined membranous and progressive glomerulonephritis or the presence of an associated second disease entity (4, 5).

The combination of anti-GBM disease and MN, first described in 1974, is rare and its treatment is documented by only a small number of case reports and series (6–9). Current research reveals several critical knowledge gaps that warrant further investigation: while the coexistence of dual nephropathies lacks standardized diagnostic criteria, the pathogenic relationship between anti-GBM disease and MN remains mechanistically unclear. Moreover, therapeutic decision-making continues to be debated due to insufficient safety data in patients with low IgG levels, and importantly, the proposed epitope spreading mechanism whereby MN-related immune complexes potentially induce anti-GBM antibody formation still requires definitive clinical evidence.

Herein, we present a case of a patient with anti-GBM disease complicated by MN. This case demonstrates significant clinical value by presenting definitive evidence supporting the concurrent diagnosis of anti-GBM disease and MN, fulfilling complete diagnostic criteria for both conditions through comprehensive clinical, pathological, and serological evaluations. The case provides systematic documentation of treatment response to a “plasmapheresis + glucocorticoid + rituximab” regimen, demonstrating the therapeutic efficacy of rituximab as a viable alternative to conventional cyclophosphamide-based immunosuppression in this dual-pathology context, particularly given the patient's low IgG status. Furthermore, the disease progression timeline and characteristic dual pathological features offer compelling clinical support for the epitope spreading hypothesis, wherein MN-related immune complex deposition potentially triggers anti-GBM antibody production. This comprehensive case report establishes an important reference for managing rare cases of coexisting immune-mediated nephropathies.

## Case presentation

This case report describes a 34-year-old male patient. A decade before the current presentation, the patient had received a diagnosis of “glomerulonephritis” without definitive characterization of clinical manifestations or laboratory findings. During that initial evaluation, renal biopsy had been recommended for pathological classification and treatment optimization, but was declined by the patient due to socioeconomic constraints and limited awareness of potential disease progression risks. Consequently, the patient failed to undergo subsequent monitoring of urinary parameters or renal function during the intervening decade. During the current hospitalization, comprehensive counseling was provided regarding the risks of chronic kidney disease progression associated with prolonged untreated glomerulonephritis, along with emphasis on the diagnostic and therapeutic urgency of the present condition. The patient and family members demonstrated full comprehension of these medical implications and provided written informed consent.

Five years earlier, he experienced proteinuria following a novel coronavirus (COVID-19) infection, with a routine urinalysis revealing protein 3+ without any signs of hematuria; however, no further treatment was conducted.

Three months prior to admission, he developed hematuria accompanied by foamy urine and occasional dysuria, bilateral pitting edema in the ankles, and facial swelling, while remaining asymptomatic for nausea, vomiting, or back pain. Ten days before admission, he presented with a fever peaking at 38.5 °C, urinalysis showed protein levels of 3+, creatinine at 114 μmol/L, and anti-glomerular basement membrane (GBM) antibodies positive. Subsequently, he sought further evaluation and was admitted for specialized care. Upon physical examination, significant pitting edema was noted in both lower limbs. He was subsequently admitted to the nephrology ward for further assessment and diagnostic investigations. Laboratory investigations revealed leukocytosis (WBC 11.85 × 10^9^/L) with neutrophilia (9.65 × 10^9^/L), nephrotic-range proteinuria (8.05 g/24h), and hypoalbuminemia (serum albumin 21.2 g/L). Renal function tests showed elevated serum creatinine (126.4 μmol/L) with profound hypogammaglobulinemia (IgG 4.85 g/L; reference 8.6–17.4 g/L). Serological workup was negative for anti-PLA2R antibodies (<2 RU/ml), ANA, ENA, and ANCA (detailed in Table 1). Ultrasound examination of the abdomen and pelvis revealed no significant abnormalities. Renal ultrasonography demonstrated preserved kidney dimensions (right 119 mm × 42 mm × 65 mm; left 117 mm × 52 mm × 56 mm), with maintained cortico-medullary differentiation but increased echogenicity. There was no evidence of obstruction or dilation of the pelvicalyceal system.

**Table 1:** Investigations of the patient upon admission.

**Test**	**Result**	**Laboratory reference range**
White blood cell	11.85	3.5–9.5 × 10^9^/L
Neutrophils	9.65	1.8–6.3 × 10^9^/L
Hemoglobin	131	130–175 g/L
Platelets	271	125–350 × 10^9^/L
Urine routine	Protein: 3+	Negative
	RBC: 21.37	0–3 per HPF
	Casts: 8.18	0–2/μl
24-h urinary protein	8.05/3,400	0–0.15 g/1,500–2,500 ml
Total protein	45.2	65–85 g/L
Albumin	21.2	40–55 g/L
Blood urea nitrogen (BUN)	6.26	2.5–7.1 mmol/L
Serum creatinine (Scr)	126.4	44–104 μmol/L
Intact parathyroid hormone (iPTH)	89.5	12–88 pg/mL
C3	1.51	0.7–1.4 g/L
C4	0.55	0.1–0.4 g/L
Immunoglobulin A	3.31	1–4.2 g/L
Immunoglobulin M	1.12	0.3–2.2 g/L
Immunoglobulin G	4.85	8.6–17.4 g/L
ANA+ENA (13 antigens)	Negative	Negative
Anti-MPO antibody	0.86	0–18 RU/ml
Anti-PR3 antibody	0.07	0–18 RU/ml
Anti-GBM antibodies measured	196.27	0–20 RU/ml
Anti-PLA2R antibodies	<2	0–20 RU/ml
FT3	2.82	3.1–6.8 pmol/L
FT4	11.11	12.8–22 pmol/L
TSH	2.5	0.27–4.2 μIU/ml

HPF, high power field; C3, complement 3; C4, complement 4; ANA, antinuclear antibodies; ENA, extractable nuclear antigens antibodies; MPO, myeloperoxidase; PR3, proteinase 3; GBM, glomerular basement membrane; PLA2R, phospholipase A2 receptor; FT3, free triiodothyronine; FT4, free thyroxine; TSH, thyroid stimulating hormone.

Renal biopsy performed on the second hospital day demonstrated characteristic immunofluorescence findings: intense granular deposition of IgG (+++), C3 (+++), and κ/λ light chains along the glomerular basement membrane (GBM), with concurrent linear IgG staining. Renal tissue anti-PLA2R antibody testing revealed strong granular positivity along the GBM. Light microscopic examination of 37 glomeruli identified one globally sclerotic glomerulus, with the majority exhibiting necrotizing and crescentic features (14 glomeruli showing crescents and/or necrosis). Additional findings included diffuse GBM thickening with silver stain-demonstrated spike formation, along with focal mononuclear interstitial infiltrates. Ultrastructural analysis confirmed irregular GBM thickening, continuous subepithelial electron-dense deposits, and extensive foot process effacement. In conjunction with positive serum anti-GBM antibodies, these histopathological features established the definitive diagnosis of concurrent anti-GBM disease and MN (as documented in the **Pathology Report**).

The patient initially presented with symptoms indicative of a mixed nephrotic and nephritic syndrome. The second day after admission, he was started on intravenous methylprednisolone at a dosage of 60 mg once daily. Following the results of kidney biopsy (the 10th day post-admission), methylprednisolone was increased to 250 mg for three consecutive days and then tapered to 40 mg daily. Starting from the 10th day post-admission, the patient also underwent seven sessions of plasmapheresis. Additionally, intravenous administration of 1.0 g of CD20 monoclonal antibody was conducted on hospital day 26, followed by an identical second dose after a 2-week interval. Supportive treatments included immunoglobulin, albumin, diuretics, low-molecular-weight heparin, prophylactic antibiotics, calcium carbonate, and calcitriol. The therapeutic approach was carefully tailored to address the patient's complex presentation of coexisting anti-GBM disease and membranous nephropathy, incorporating critical clinical parameters into the decision-making process. Considering the life-threatening nature of anti-GBM disease, as evidenced by the markedly elevated antibody titer (196.27 RU/ml) and renal impairment (serum creatinine 126.4 μmol/L), immediate initiation of plasmapheresis combined with high-dose methylprednisolone pulse therapy was imperative to achieve rapid antibody removal and immune suppression. No adverse reactions occurred during the entire course of treatment. Concurrently, the management of membranous nephropathy necessitated targeted immunomodulation of B-cell-mediated pathogenesis, for which rituximab was specifically selected given its dual capacity to address both disease processes while mitigating the risks associated with conventional immunosuppressive regimens.

**Figure 1:**
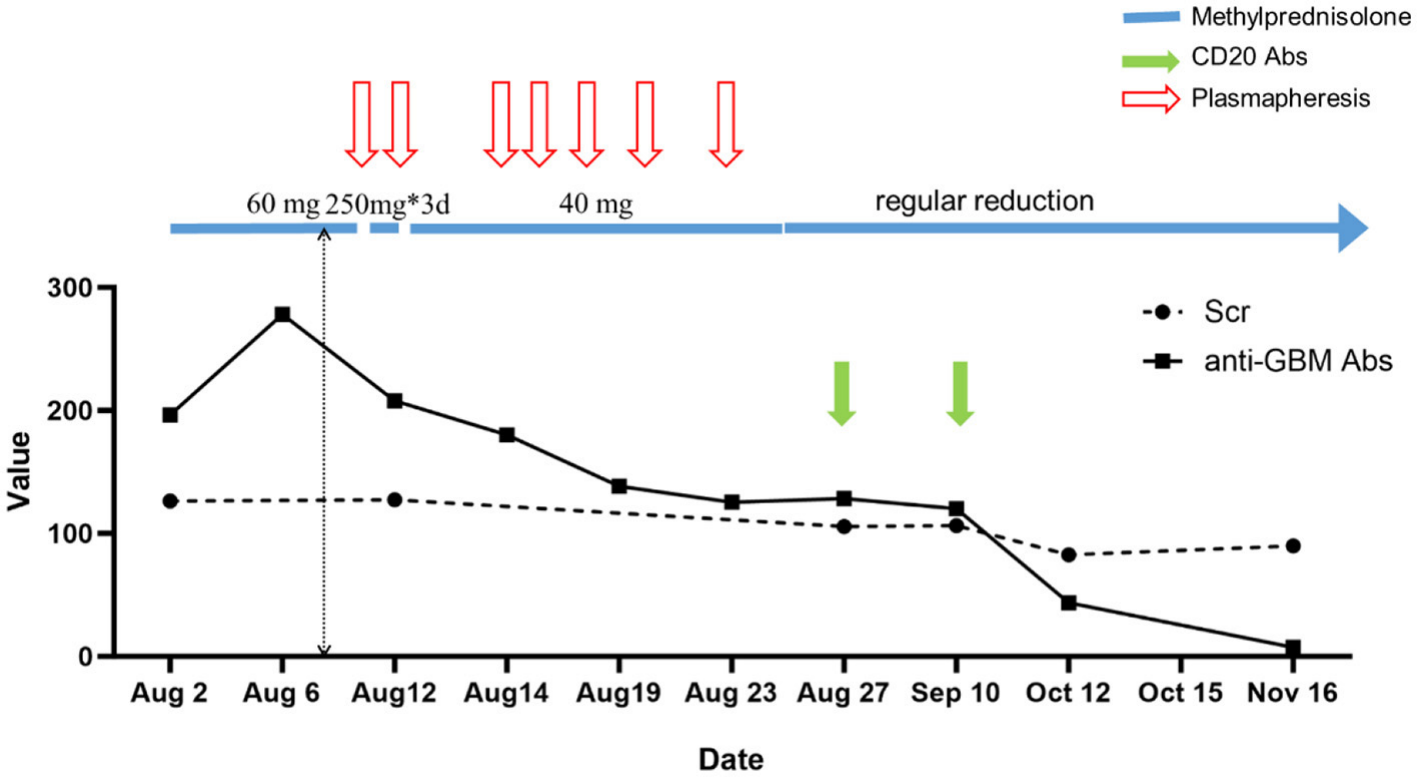
Clinical course. Scr, serum creatinine; anti-GBM Abs, anti-glomerular basement membrane antibody.

Subsequent tapering of methylprednisolone was performed. After 4 weeks of hospitalization, the patient's overall condition improved, with no resolution of edema and no abnormalities in heart, lungs, or abdomen. Eventually, blood creatinine levels returned to normal, and the anti-GBM antibodies were negative. The patient's kidney function recovered, and renal replacement therapy was not required (shown in Figure 1).

## Pathology report

**Figure 2:**
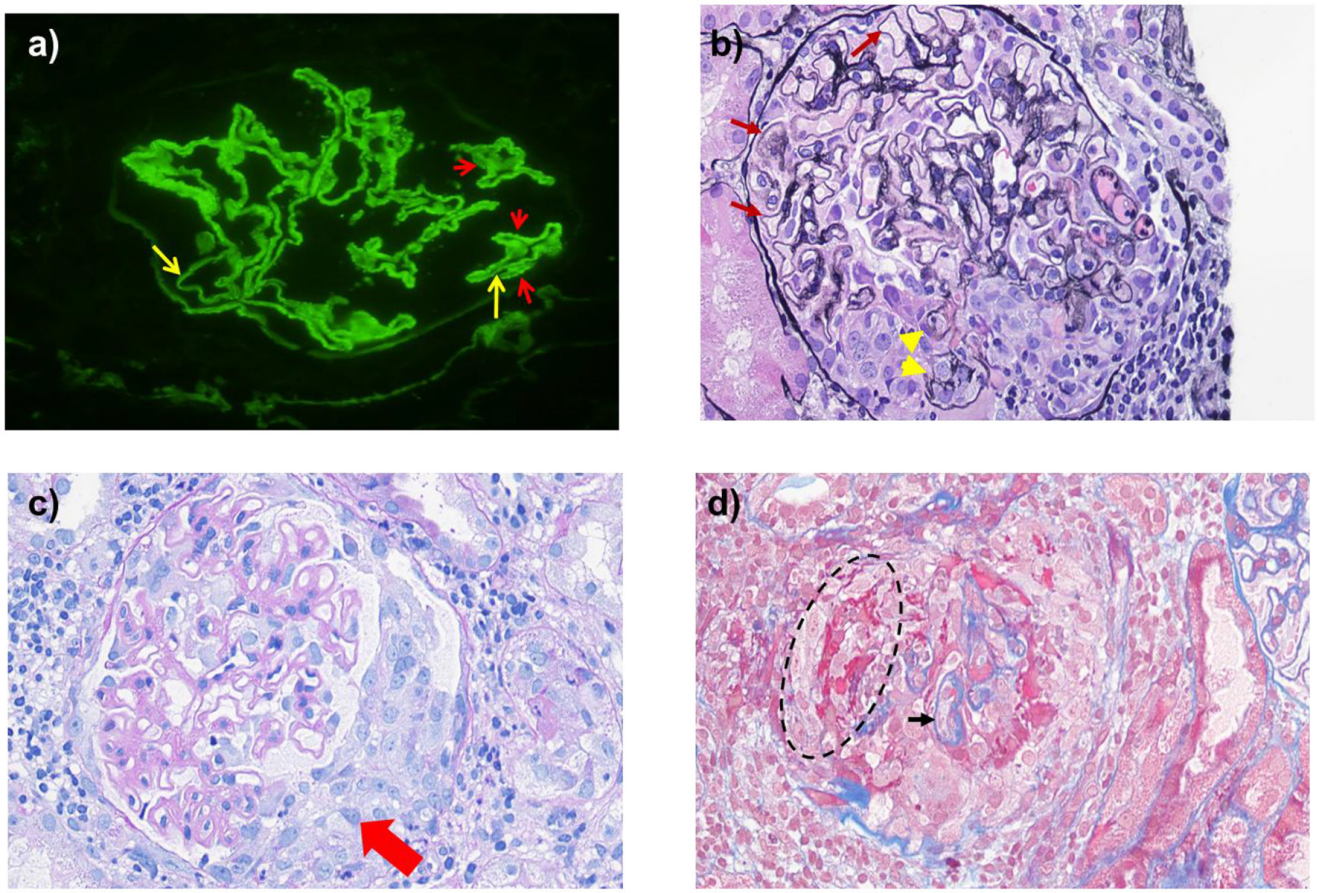
Kidney biopsy. **(a)** Granular (red arrows) and linear (yellow arrows) IgG deposition along the glomerular basement membrane (immunofuorescence, × 400); **(b)** A diffuse and moderate thickening of glomerular basement membrane with segmental ruptures (yellow arrows), and diffuse spike-like changes (red arrows)in the GBM (PASM, × 400); **(c)** cellular crescents (red arrows; PAS, × 400); **(d)** Granular eosinophilic deposits are observed on the residual outer side of the GBM (black arrow) in the context of large cellular crescents and fibrinoid necrosis (black cycle) (Masson's staining, × 400).

Immunofluorescence analysis revealed the following: IgA (–), IgG (+++), IgM (–), C3 (+++), C1q (–), κ (+++), λ (+++), IgG1 (++), IgG2 (–), IgG3 (–), IgG4 (++). There was strong granular positivity along the GBM, with observable linear positivity against this background and minimal linear deposition noted in some tubular basement membranes (TBMs) (shown in Figure 2a). Light microscopy identified two fragments of cortical kidney tissue containing a total of 37 glomeruli, including one globular waste (shown in Figure 2). The majority of glomeruli exhibited necrosis and crescents. Six with cellular crescents and four with large cellular crescents, while four glomeruli were completely necrotic, characterized by extensive rupture of the Bowman's capsule and surrounding inflammatory cell infiltration, leaving only minimal residual glomerular tuft (shown in Figure 2c). Eight glomeruli exhibited either segmental ruptures of the GBM or the Bowman's capsule, with or without fibrinoid exudation. Although no proliferative lesions were observed, the GBM exhibited diffuse moderate thickening and stiffness, with neutrophils present in some segments of the loop cavities, while other cavities remained patent. Masson's staining demonstrated abundant, diffuse granular hemosiderin deposition subepithelially, while silver staining revealed diffuse spike-like changes in the GBM. The renal tubules showed no signs of atrophy, however, some epithelial cells exhibited granular degeneration. A few tubules appeared slightly wrinkled, with widened interstitial spaces and varying degrees of brush border loss in several epithelial cells. Individual tubules displayed TBM rupture accompanied by tubular inflammation, sparse protein casts and red blood cell casts. The interstitium exhibited significant focal infiltration of mononuclear inflammatory cells, neutrophils, and occasional eosinophils, along with mild focal edema and slight fibrosis. No significant sclerotic changes or evidence of arteritis or necrosis were observed.

**Figure 3:**
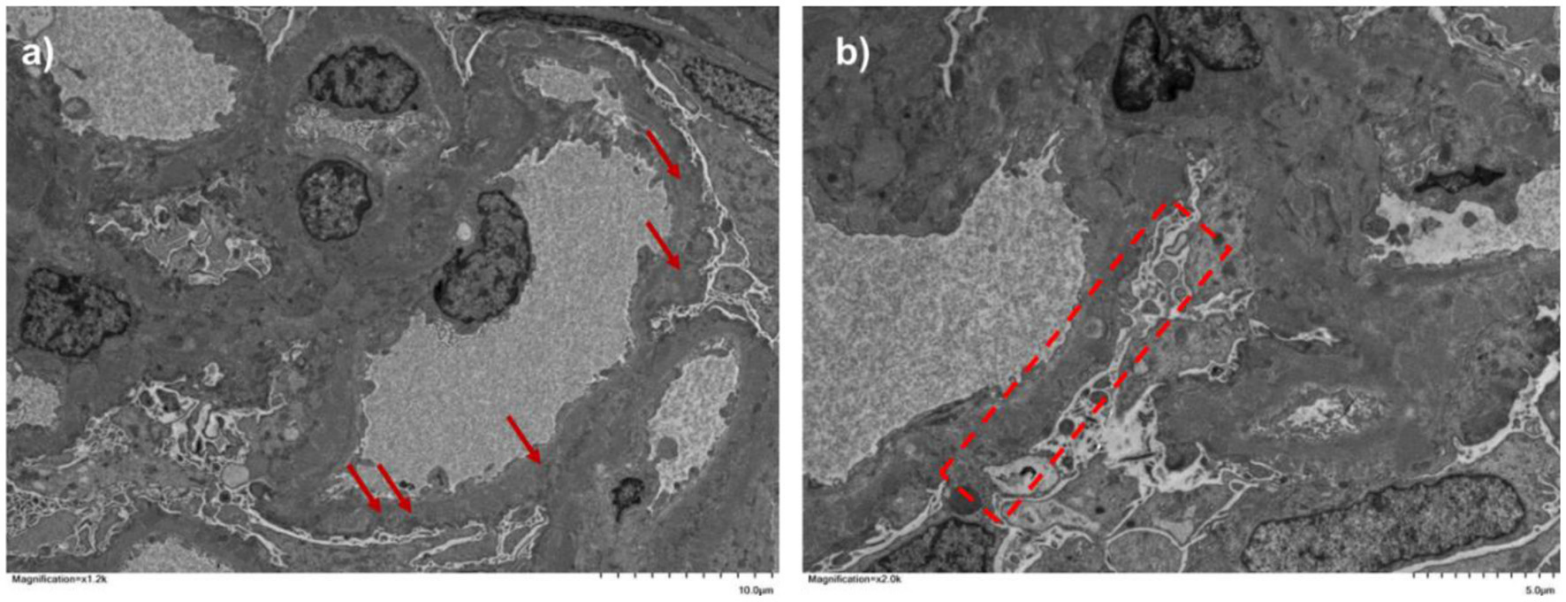
Kidney biopsy. Subepithelial electron-dense deposits [**(a)**, ×12,000] and diffused podocyte foot-process detachment [**(b)**, ×20,000].

The electron microscopy report was finalized, revealing one glomerulus. The capillary loops appeared stiff, and there was no significant proliferation of podocyte. The capillary basement membrane exhibited irregular thickening, accompanied by a relatively continuous deposition of electron-dense substances beneath the epithelial cells, some deposits migrated into the membrane while others partially dissolved. Diffuse fusion of the foot processes was also observed. Mild proliferation of mesangial cells and an increase in the mesangial matrix was noted, although no significant deposition of electron-dense substances occurred in the mesangial area. The renal interstitium displayed scattered inflammatory cells and deposits of bundled collagen fibers (shown in Figure 3).

## Discussion

Anti-glomerular basement membrane (anti-GBM) disease occasionally co-exists with a second disease, rarely in conjunction with membranous nephropathy (MN). In 1974, Klassen et al. were the first to report the co-occurrence of anti-GBM disease and MN. Subsequently, Jia et al. analyzed eight patients with anti-GBM disease and MN, exhibited a male predominance, and none presented with oliguric or anuric condition, a lower level of serum creatinine (Scr) levels at diagnosis, resulting in significantly better kidney prognosis after 1 year of follow-up when compared to patients with anti-GBM disease alone (kidney recovery rates of 62.5% vs. 13.3%) (6). Furthermore, Zhang et al. also found that only 14.3% of anti-GBM and MN patients had crescentic GN and an average necrosis of 29.0%, revealing a better renal function prognosis than those with classical anti-GBM GN group (8). One the other hand, Ahmad et al. reported that progression to a requirement for kidney replacement therapy occurred in all 12 patients with anti-GBM disease and MN, only 2 (16.7%) later recovered kidney function (7). Bu et al. reported the clinic-pathologic characteristics and outcome of 28 patients with this dual glomerulopathy. In this cohort, 57% were male, and the median age at diagnosis was 54 years (range 15–82), a total of 96% presented with acute kidney injury, with a median serum creatinine at biopsy of 7.8 mg/dl (range 1.2–24.0), along with proteinuria (median 3.5 g/d, range 0.4–11) and hematuria. After a median follow-up of 17 months (range 0.3–248), 3 patients (11%) achieved complete remission, 7 (27%) experienced persistent kidney dysfunction, and 16 (62%) progressed to end-stage kidney disease (ESKD) (10).

MN, a prevalent glomerulopathy, exhibits a core pathogenic mechanism involving subepithelial immune complex deposition along the GBM and complement system activation, ultimately resulting in podocyte injury and filtration barrier dysfunction. While PLA2R represents the first identified specific target antigen, non-PLA2R-associated MN (as exemplified by this anti-PLA2R antibody-negative case) may involve alternative target antigens (e.g., THSD7A) or undefined immune complexes, yet consistently demonstrates the hallmark pathological features of chronic GBM damage and persistent immune complex deposition (11, 12). Importantly, immunofluorescence patterns—particularly the predominant granular IgG4 deposition along the GBM (with occasional IgG1 predominance) frequently accompanied by C3—serve as critical diagnostic discriminators (13, 14). Recent advances in MN pathogenesis research have revealed that sirtuin 6 confers direct podocyte protection through Wnt1-β-catenin pathway inhibition to suppress renin-angiotensin system activation, while gut microbial metabolites have been implicated in renal pathophysiology via complex regulatory networks contributing to MN development, collectively underscoring the necessity for multidimensional therapeutic strategies in MN management (15–17).

The patient in this case is a middle-aged male who presented with proteinuria, followed by glomerular hematuria and kidney damage. The patient's clinical progression demonstrates a distinct pathogenic sequence wherein MN clearly antedated the development of anti-GBM disease. The initial manifestation of proteinuria, characteristic of MN, marked the inception of the disease process, while the subsequent emergence of hematuria and renal dysfunction corresponded precisely with the progressive features of anti-GBM disease. The renal histopathology demonstrated coexisting pathognomonic features of MN (subepithelial electron-dense deposits) and classic anti-GBM disease (crescentic formations), while electron microscopy revealed distinct spike-like projections of the GBM without evidence of disruption. These findings are fundamentally incompatible with the expected pathological manifestations of primary anti-GBM disease, in which untreated cases would exhibit acute inflammatory necrosis causing structural disintegration of the GBM, precluding the formation of spike-like projections and invariably demonstrating GBM rupture on ultrastructural examination, typically accompanied by renal atrophy. The pathological mechanism may involve the deposition of immune complexes on the epithelial side if the GBM, causing damage and exposing the concealed α3 chain of type IV collagen [α3(IV)NC1] epitopes, which induce the production of anti-GBM antibodies, leading to crescent formation and deterioration of renal function (18). Notably, in patients with both anti-GBM disease and MN, anti-PLA2R antibodies are largely negative, with only a few reported cases that are positive. Some studies have confirmed the correlation between the anti-GBM disease and MN. Antibodies were detectable in circulation 2 weeks after immunization with α3(IV)NC1, with the onset of proteinuria persisting for 8 to 10 weeks, accompanied by renal pathology indicative of MN. Moreover, six of 10 patients with anti-GBM disease complicated with a well-documented glomerulonephritis, including MN, which reflecting that the immune complexes deposited in glomeruli might participate in the initiation of anti-GBM disease (19). The proposed mechanism involves anti-GBM antibodies inflicting damage to podocytes, thereby increasing GBM permeability and concurrently releasing antigens that form immune complexes, ultimately resulting in immune complex nephritis. At the genetic level, the HLA-DRB1 and HLA-DQ1 alleles are closely associated with susceptibility to anti-GBM disease and MN, the coexistence of these alleles may increase the risk of comorbidities, for example, HLA allele DRB1^*^15:01 (20).

Currently, reports of MN complicated by anti-GBM disease primarily consist of case studies, with treatment regimens mirroring those for classical anti-GBM disease, including steroid, immunosuppressant (for example, cyclophosphamide/CTX), and plasmapheresis. The patient in our case received a high dose of methylprednisolone, plasmapheresis, and CD20 monoclonal antibody, achieving renal recovery, alongside a reduction in urinary protein and a favorable kidney prognosis. The avoidance of cyclophosphamide was warranted given its dual risks of exacerbating bone marrow suppression—thereby compounding infection susceptibility in this immunocompromised patient—and its well-documented gonadotoxic effects that carry particular significance for reproductive-age male patients. The avoidance of cyclophosphamide was warranted given its dual risks of exacerbating bone marrow suppression—thereby compounding infection susceptibility in this immunocompromised patient—and its well-documented gonadotoxic effects that carry particular significance for reproductive-age male patients (21). Conversely, rituximab emerged as the preferable therapeutic alternative through its selective B-cell depletion mechanism, which not only demonstrates established efficacy in ameliorating proteinuria in membranous nephropathy but also exhibits synergistic effects with plasmapheresis and glucocorticoids by enhancing pathogenic antibody clearance in anti-GBM disease associated with autoimmune pathogenesis (22). Furthermore, clinical studies have demonstrated that cyclophosphamide not only significantly improves rates of complete clinical and immunological remission, but also reduces relapse risk while minimizing potential complications associated with prolonged immunosuppressive therapy (23). Liu et al. reported that the use of plasmapheresis was independently associated with a reduced risk of primary outcome (OR: 0.179), better 2-year (HR: 0.146) and 8-year patient survival (HR: 0.309). Patients who initiated plasmapheresis early had a better prognosis and might only need 5–10 plasmapheresis sessions to achieve maximal risk reduction (24). B-cell depletion with anti-CD20 monoclonal antibodies is widely used for the treatment of autoimmune diseases, including MN, lupus, ANCA-associated glomerulonephritis and so on. Currently, the application of rituximab in anti-GBM disease is primarily documented through case reports (25). B cell monoclonal antibodies could effectively reduce anti-GBM antibody levels, decrease the necessity for plasmapheresis, and promote disease remission. Early detection, in conjunction with plasmapheresis, steroids, and immunosuppressive therapy, may result in positive outcomes.

This study has several limitations that warrant consideration. First, as a single case report, the findings may not be generalizable to all patients with this dual glomerulopathy, necessitating validation through larger cohort studies to confirm the observed clinical and pathological characteristics. Second, the limited duration of follow-up precludes definitive conclusions about the durability of renal function recovery and potential relapse risks, which require longer-term observation. Third, the specific target antigen responsible for PLA2R-negative MN in this case remains unidentified, as comprehensive testing for other MN-associated antigens (e.g., THSD7A, NELL-1) was not performed, potentially constraining our understanding of the triggering factors for anti-GBM disease in this context. Future investigations with larger sample sizes and extended follow-up periods are needed to elucidate the pathogenesis of this complex disease entity and optimize therapeutic strategies.

## Conclusion

Anti-GBM and MN is clinically rare and presents unique characteristics in terms of clinical presentations, renal pathology, and immunological examinations. By integrating tests for anti-GBM antibodies, anti-PLA2R antibodies, and renal pathology evaluations, early diagnosis becomes feasible. For patients presenting with persistent proteinuria followed by acute hematuria and renal dysfunction, clinicians should maintain a high index of suspicion for potential concurrent membranous nephropathy and anti-GBM disease. Comprehensive integration of serological markers and renal pathological examination enables precise differentiation. In young male patients with hypogammaglobulinemia, preferential use of B-cell targeted therapies such as rituximab offers dual advantages of maintained therapeutic efficacy while circumventing the safety concerns associated with cyclophosphamide, thereby providing valuable insights for personalized management of complex glomerular diseases. Furthermore, early administration of glucocorticoids combined with plasmapheresis, and CTX or CD20 antibodies can be beneficial for the recovery of renal function.

## Data Availability

The raw data supporting the conclusions of this article will be made available by the authors, without undue reservation.
